# Expression of recombinant human coagulation factors VII (rFVII) and IX (rFIX) in various cell types, glycosylation analysis, and pharmacokinetic comparison

**DOI:** 10.1186/1753-6561-5-S8-P23

**Published:** 2011-11-22

**Authors:** Ernst Böhm, Michael Dockal, Michael Graninger, Meinhard Hasslacher, Martin Kaliwoda, Christian Konetschny, Artur Mitterer, Eva-Maria Muchitsch, Manfred Reiter, Friedrich Scheiflinger

**Affiliations:** 1 Baxter BioScience, Orth/Donau, A-2304, Austria; 2 Baxter BioScience, Vienna, A-1220, Austria

## Introduction

Clearance mechanisms for rFVII (or the active enzyme rFVIIa) and rFIX are influenced by post-translational modifications, especially N-glycosylation. This should be considered when choosing a recombinant expression system in view of the varying ability of frequently used cell lines to perform modifications similar to human proteins.

Differences in the pharmacokinetic properties of recombinant FVIIa versus plasma-derived (pd)FVII or desialylated rFVIIa are known for human FVII(a). Asialo rFVIIa clears quickest, whereas pdFVII, having a higher degree of sialylation, is cleared to a lesser extent [1]. In the case of FIX, the degrees of serine phosphorylation and tyrosine sulfation in the activation peptide have been postulated to influence pharmacokinetic behavior, especially *in vivo* recovery [2].

We chose CHO, BHK and HEK293 cells for expression of rFVII to compare post-translational protein modifications, and HEK293-derived cell lines to generate highly phosphorylated and sulfated rFIX for *in vivo* studies. rFIX from the same clone and production run was purified using two different down-stream processes: The first to enrich high phosphorylated and sulfated protein, the second to purify total rFIX at high yield. These HEK293-derived rFIX isoforms were compared with CHOrFIX and pdFIX in a pharmacokinetic study in FIX knock-out mice.

## Materials and methods

### Proteins

pdFVII and pdFIX were from HTI, recombinant, CHO-derived FIX was from Wyeth.

### Cell culture

BHK (BHK-21; ATCC#CCL-10™) and HEK293 cells (ATCC#1531) were from American Type Culture Collection, CHO DXB11 from University of Columbia. All were cultivated in DMEM/Ham’s F12 medium containing fetal bovine serum (FBS). BHK and HEK293 cell-derived rFVII producer clones were selected by antibiotic resistance. CHO DXB11-derived producer clones were generated by methotrexate gene co-amplification. rFVII was produced in vitamin K-containing medium without FBS.

HEK293 cells producing human FIX were selected by antibiotic resistance. Clones were adapted to serum-free suspension culture in Excell293 medium containing vitamin K, and cultivated in repeated batch mode fermentation runs.

### Purification

rFVII was purified using Q-Sepharose FF for capture, and a tandem step containing a cellufine sulfate column connected to Q-Sepharose FF.

HEK293-derived rFIX from the same clone from one fermentation run was purified from culture supernatants with two different purification schemas: for high yield, rFIX was loaded in the presence of EDTA on Q-Sepharose FF, the column was washed with EDTA at high salt concentration, FIX was eluted at low salt CaCl_2_. For enrichment of the highly phosphorylated and sulfated protein fraction, rFIX was loaded in the presence of EDTA on fractogel TMAE, washed with EDTA at high salt concentration, and eluted with a salt gradient in the presence of CaCl_2_.

### Analytics

Purified FVII forms were analyzed using reversed phase HPLC, LC-MS for intact protein or peptide mapping after trypsin digestion to confirm sequences and post-translational modifications, and anion exchange HPLC for monosaccharide composition, sialic acid quantification, and comparison of N-glycans.

Degrees of FIX-phosphorylation/sulfation were monitored by LC-MS. N-glycosylation was characterized by oligosaccharide mapping (HPLC method).

### In vivo pharmacokinetic study

FIX preparations were administered to FIX-knock-out mice intravenpusly at a nominal dose of 75 U/kg. Citrate plasmas were obtained by heart puncture. FIX levels in plasmas were determined by antigen enzyme-linked immunoassay and by activated partial thromboplastine time (APTT) clotting assay.

## Results and conclusions

Identical protein structures and minor differences in post-translational modifications between rFVII from BHK, CHO, HEK293 cells, and pdFVII were found, except for N-glycosylation. Protein activities of rFVII determined by antigen and activity assays were similar for each cell type. Most N-glycans of pdFVII are bi- and triantennary, non-fucosylated structures with almost complete sialylation [3]. In contrast, all rFVII forms were sialylated only in part. Approximately 50 % N-glycans of BHKrFVII were composed of a core-fucosylated biantennary complex-type with two terminal sialic acids. Another 20 % contained terminal GalNAc, a sugar moiety with a high affinity for the asialoglycoprotein receptor involved in the *in vivo* clearance of glycoproteins from circulation. GalNAc were not found on N-glycans on CHO-derived rFVII. Most oligosaccharides on CHOrFVII were core fucosylated, biantennary structures carrying two sialic acid residues. HEK293rFVII showed major differences from all other rFVII forms, and, unexpectedly, from pdFVII. Most oligosaccharide structures were not comparable to those published for pdFVII [3], or found on rFVII from the other cell lines. A high content of fucosylation and terminal GalNAc, and a lower degree of terminal sialic acids were measured, and tri- or tetra-antennary structures were not found. Some oligosaccharides were of a hybrid type with high-mannose structures. These N-glycan structures do not favor the use of HEK293 cells as a production platform for human biotherapeutics, when protein recovery and half-life in the circulation are of importance, and influenced by N-glycosylation.

**Figure1:**
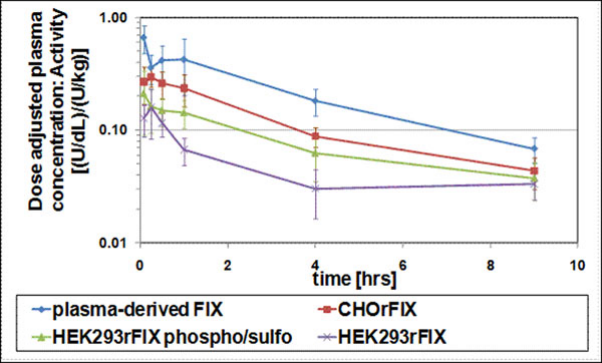
Pharmacokinetic comparison of pdFIX, CHOrFIX, HEK293rFIX, and high-phospho/sulfo HEK293rFIX in FIX-knock-out mice. Plasma concentrations of FIX after administration are shown as mean values ± standard deviations with n = 10 mice for each timepoint. Dose adjustment was done by normalizing to start material concentrations.

Pharmacokinetic differences between pdFIX and CHO-derived rFIX were as expected from the literature; HEK293rFIX materials (total rFIX and high phospho/sulfo rFIX) were both inferior to CHO-derived rFIX in terms of area under the curve (AUC) (Table 1) and *in vivo* recovery. Similar terminal half-lives and mean residence times, but different *in vivo* recoveries between rFIX and pdFIX preparations were confirmed by statistical analysis. High phospho/sulfo rFIX from HEK293 had a larger AUC and *in vivo* recovery than total rFIX from the same HEK293-derived clone and fermentation run, indicating an influence of these modifications on pharmacokinetics (Figure 1). Oligosaccharide mapping showed high glycosylation heterogeneity for HEK293 and pdFIX, and more defined peak-groups representing mono- to tetrasialylated glycans for CHO-derived material. Sialic acid content of N-glycans was 25 % lower for HEK293- than for CHOrFIX. Glycosylation and phosphorylation/sulfation degrees contributed to pharmacokinetics of FIX preparations. If glycosylation was similar in case of the two HEK293rFIX preparations, the effects of phosphorylation and sulfation became evident.

**Table 1:** AUCs of pdFIX, CHOrFIX, HEK293rFIX, and high-phospho/sulfo HEK293rFIX in a FIX-knock-out mouse pharmacokinetic study. Only relative AUC values are shown. Statistically significant different ratios to CHOrFIX (at the 5 % level) are marked with an asterisk*. The ratio of HEK293rFIX high phospho/sulfo to HEK293- total rFIX was also statistically significantly different for both antigen and activity.

	Relative value of AUC: antigen	Relative value of AUC: clotting activity
**pdFIX**	1.5*	1.8*
**CHOrFIX**	1	1
**HEK293rFIX high phospho/sulfo**	0.7*	0.7*
**HEK293rFIX**	0.4*	0.4*

In conclusion, these data showed that HEK293 cells were not adequate for rFIX or rFVII production due to improper N-glycosylation compared with the material from other cell lines, or from human plasma. Consequently, an advantage of this human cell line for the production of “more-human-like” biotherapeutics could not be observed.
